# Peptidoglycan LD-Transpeptidases

**DOI:** 10.3390/antibiotics14121210

**Published:** 2025-12-01

**Authors:** Samuel Gastrell, Waldemar Vollmer

**Affiliations:** Centre for Superbug Solutions, Institute for Molecular Bioscience, The University of Queensland, Brisbane 4072, Australia; s.gastrell@uq.edu.au

**Keywords:** LD-transpeptidase, peptidoglycan, resistance, antibacterials, cell envelope, β-lactam, bacteria

## Abstract

LD-Transpeptidases (LDTs) are a widely conserved class of peptidoglycan (PG) crosslinking enzymes in bacteria. They are sometimes overlooked as they often act secondary to penicillin binding proteins (PBPs) under standard conditions. However, LDTs are essential in key pathogens such as *Clostridioides difficile* and are responsible for β-lactam resistance in *Mycobacterium tuberculosis* and *Enterococcus faecium* due their low affinity for penicillins and cephalosporins, allowing them to form LD-crosslinks when DD-crosslinking PBPs are inactivated. This role makes LDTs a promising target when developing new treatments for these pathogens. LDTs can perform different enzymatic reactions. Most commonly they reinforce the PG with 3,3-LD-crosslinks or, in a few cases, 1,3-LD-crosslinks, during stationary phase or stress responses. Some LDTs also incorporate endogenous and exogenous non-canonical D-amino acids into the PG. In many Gram-negative bacteria, specialised LDTs tether lipoproteins or outer membrane proteins (OMPs) to the PG to maintain cell envelope integrity; in some cases this regulates virulence factors. Specialised LDTs have also been implied to have roles in polar growth, toxin secretion, and symbiotic colonisation. Recent discoveries include novel subgroups of the major YkuD family and the identification of the VanW family; this has opened new research directions surrounding LDTs. We aim to understand LDTs and their roles to expand our knowledge of PG synthesis and modification and how these enzymes can be targeted for antibiotic treatment.

## 1. Introduction

**Figure 1:**
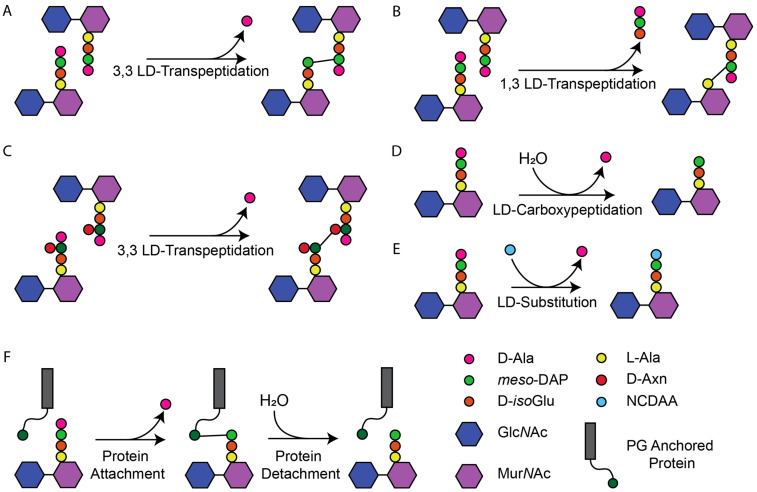
Representation of reactions catalysed by LDTs. (**A**) Formation of 3,3 *meso*-DAP-*meso*-DAP LD-crosslinks typical in Gram-negative bacteria. (**B**) 1,3-LD TPase reactions observed in *Gluconobacter oxydans* cleaving L-Ala-D-*iso*Glu of the donor peptide to form L-Ala-*meso*-DAP. (**C**) Formation of L-Lys-D-Asx-L-Lys crosslinks observed in *E. faecium*. (**D**) LD-CPase activity resolves the donor acyl-enzyme complex with water (H_2_O) as the acceptor substrate (hydrolysis). (**E**) Substitution of D-Ala with a non-canonical D-amino acid (NCDAA) acting as acceptor substrate. (**F**) Membrane protein attachment and detachment. During attachment the proteins acts as the acceptor substrate to the tetrapeptide donor. During detachment, hydrolysis of the linkage is catalysed by YkuD family amidases.

Peptidoglycan (PG) is an essential component of the bacterial cell envelope. It forms a net-like macromolecule around the cytoplasmic membrane (CM) and is needed by bacteria to prevent lysis due to their turgor and to maintain cell shape [1,2]. PG consists of glycan chains of alternating *N*-acetylglucosamine (Glc*N*Ac) and *N*-acetylmuramic acid (Mur*N*Ac) joined by peptides bound to the lactyl group of Mur*N*Ac [1,2,3]. The nascent peptides consist of five amino acids. In Gram-negative bacteria, the most common sequence is L-Ala-D-*iso*-Glu-*meso*-diaminopimelic acid (*meso*-DAP)-D-Ala-D-Ala. In Gram-positive bacteria, the peptides are more variable at positions two and three with D-*iso*Gln and L-Lys being the most common. The synthesis of PG from lipid II requires PG synthases to polymerise the glycan strands by glycosyltransferases (GTase) and crosslink the peptides by transpeptidases (TPase). Peptides from adjacent glycan chains can be crosslinked with either DD or LD bonds formed by penicillin-binding proteins (PBPs) and LD-Transpeptidases (LDTs), respectively. Both release a terminal D-Ala residue during the transpeptidation reaction [1,4]. PBPs require a pentapeptide donor and form crosslinks between the fourth residue of one peptide and the third of another (4,3-DD-crosslinks). LDTs require a tetrapeptide donor and form LD-crosslinks between position three of a peptide and three of another (3,3-LD crosslinks) or position one of a peptide and three of another (1,3-LD-crosslinks) (Figure 1) [1,2,5,6,7]. DD-crosslinks are the predominant crosslinks in most species, formed during PG synthesis for growth and cell division [2,3]. LD-crosslinks are often formed in mature PG as part of cell wall repair, stress responses, or antibiotic resistance mechanisms [1,7,8]. While crosslinking is the most common reaction, LDTs have a wider range of reactions they catalyse (Figure 1), not all of which are LD bonds as seen in the protein attachment and certain substitution reactions [9,10,11,12,13]. Hence, LDTs are defined by the LD *meso*-DAP-D-Ala bond they break rather than the bonds they form [14,15].

There are two families of LDTs, YkuD and VanW [7,16]. The YkuD family was discovered in the year 2000 in ampicillin-resistant *Enterococcus faecium* [7,17]. YkuD-family LDTs display LD-transpeptidase (LD-TPase), LD-carboxypeptidase (LD-CPase), or LD-endopeptidase (LD-EPase) activity in a range of different roles such as crosslinking PG, anchoring proteins to or detaching them from the PG, or incorporating non-canonical D-amino acids (NCDAAs) (Figure 1) [2,18,19,20,21,22,23]. YkuD proteins are widely conserved across Gram-negative and Gram-positive bacteria with >123,000 homologs identified across >30,000 species by the PFAM database (15 September 2025) [16,24,25]. The VanW family proteins were first recorded as unknown proteins associated with vancomycin resistance [26]. Their activity was identified in 2024 as essential LDTs forming crosslinks in *Clostridioides difficile* [16]. Some VanW proteins display no LD-TPase activity and their roles remain unknown [16]. VanW proteins are less abundant than YkuD proteins, with members mostly restricted to Gram-positive species. Currently >14,000 VanW homologs are known across >9000 species, according to the PFAM database (15 September 2025) [16,24,25].

The formation of LD-crosslinks in the PG can increase the degree of overall crosslinking, which is believed to strengthen the PG [5,27]. This is supported by their common association with stringent and envelope stress responses in many species [5,8,18,27,28,29,30,31]. They are also constitutively active in several species with highly crosslinked cell walls where they provide resistance to many environmental stresses [16,29,32,33]. YkuD family LDTs display low affinity for penicillin and cephalosporin class β-lactams facilitating resistance in key pathogens such as *Mycobacterium tuberculosis*, *E. faecium*, and *C. difficile* making them promising antibiotic targets [7,27,31,34,35,36].

LDTs covalently attach membrane-anchored proteins to the PG in Gram-negative species to maintain cell envelope integrity and the widths of the periplasm [23,37,38]. Braun’s lipoprotein (Lpp) in *Escherichia coli* was the first known bacterial lipoprotein and PG-attached protein [9,38,39,40]. In recent years, other OM-anchored lipoproteins, outer membrane beta-barrel proteins (OMPs), and one CM protein have been identified anchored to PG [10,23,37,39,41,42]. The recently identified YkuD family amidase DpaA detaches Lpp from PG, showing that the Lpp-PG binding is dynamic [19,43,44].

Lastly, some bacteria have developed specialised ways to utilise LDTs including toxin release in Type 10 secretion systems (T10SS) [45,46], symbiotic colonisation [47], repair of PG damage [8], and lysozyme resistance [48].

LDTs can be utilised to incorporate probes into PG, which can be visualised by electron and fluorescence microscopy [49]. Visualisation of biotinylated D-Cys and fluorescent D-amino acid probes have provided key insight into the growth pattern of the PG sacculus in different bacteria [49,50,51]. Beyond this, LD-TPase specific fluorescent probes have recently been developed to better understand LDT activity [49,50,52].

In this review we will investigate the different classes of LDTs and what is known of their roles in species and how these roles vary across related bacteria. We will also highlight what is known about their use as antibiotic targets and identify new avenues for antibiotic research.

## 2. The Structure and Mechanism of LDTs

LDT activity was initially observed in vitro with *Enterococcus faecalis* membranes as an activity that substituted the terminal D-Ala in Ac_2_-L-Lys-D-Ala for environmental NCDAAs [53]. At a similar time, in vivo LD-crosslinks were discovered in the cell wall of *Mycobacterium smegmatis* and these comprised ~33% of the total crosslinks [5]. NCDAA incorporation into the PG of growing cells was first observed in *E. coli* [54,55].

The first LDT identified was the YkuD family Ldt_fm_ of *E. faecium* [17]. A mutant with a defective PBP5 displayed β-lactam resistance facilitated by a PG consisting of only LD-crosslinks [17]. Further investigations into this novel mechanism of antibiotic resistance lead to the identification of the key Cys-His-small residue catalytic triad, and the structure of the LDT was subsequently solved from using crystallography [56,57].

**Figure 2:**
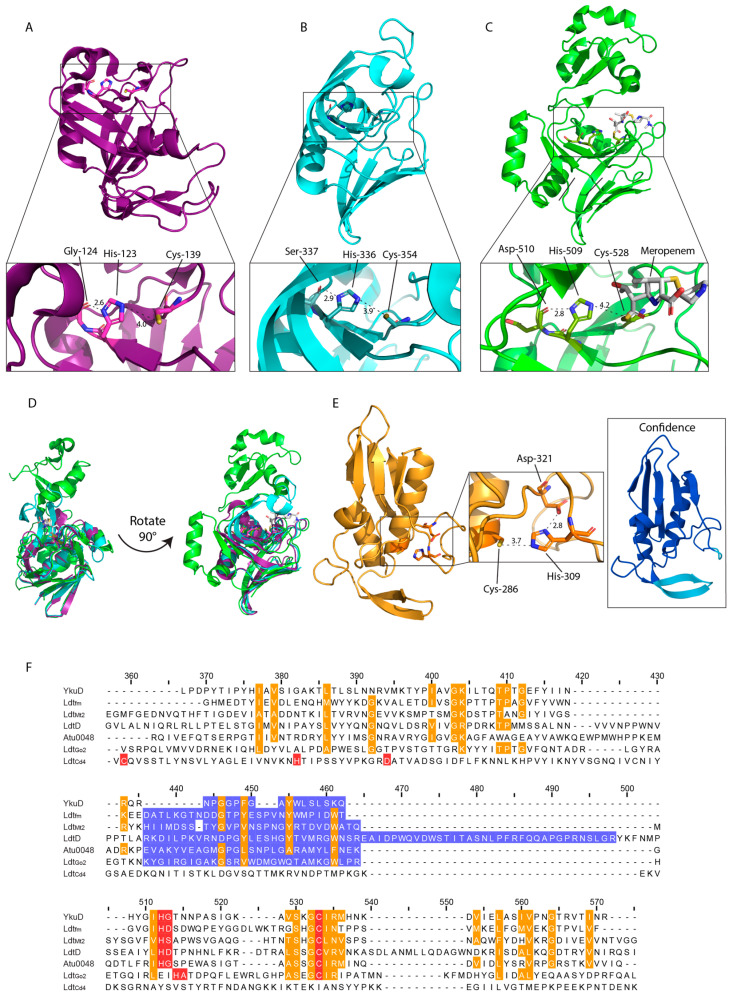
Structures of the catalytic domains of YkuD-type LDTs. (**A**) Structure of the catalytic domain of YkuD from *B. subtilis* (PDB: 1Y7M res 55–164 [24]). (**B**) Structure of the catalytic domain of Ldt_Mt2_ from *M. tuberculosis* (PDB: 5DU7_C res 252–379 [62]). (**C**) Structure of the catalytic domain of LdtD from *E. coli* (PDB: 6NTW res 375–576 [58]). (**D**) YkuD catalytic domain with catalytic domains from Ldt_Mt2_ (TM-score = 0.57) and LdtD (TM-score = 0.60) overlayed [61]. (**E**) Alphafold [63] representation of the catalytic domain and active site of Ldt_Cd4_ from *C. difficile* (VanW-type) (CBE03724.1 res 228–357 [16]). Confidence presented as blue–yellow gradient with blue most confident and yellow least confident. (**F**) Sequence alignment of active site residues of LDT catalytic domains from various species including YkuD family *B. subtilis* YkuD, *E. faecium* Ldtfm, *M. tuberculosis* Ldt_Mt2_, *E. coli* LdtD, and *Agrobacteria tumefaciens* Atu0048, the YkuD-like *G. oxydans* Ldt_Go2_ and VanW family *C. difficile* Ldt_Cd4_. Catalytic triad residues in red, conserved residues in orange, variable capping loop region in blue.

The structure of Ldt_fm_, alongside YkuD in *Bacillus subtilis*, lead to the characterisation of the catalytic domain termed YkuD. This domain consists of a β-sandwich of two five-six strand mixed β-sheets joined by two hinge α-helices with activity, which is facilitated by the catalytic triad (Figure 2A) [24,57]. The YkuD-fold contains the sequence ϕGϕHG(S/T)-(X)_10_(S/T)XGCϕR(M/L), where ϕ is a hydrophobic residue and X denotes any amino acid, which is conserved across various LDTs from Gram-negative and Gram-positive species (Figure 2F) [24]. The structure of the YkuD-domain is also conserved, forming a groove with two cavities, inner and outer, which provide access to the catalytic site. The exception is the variable capping loop region (Figure 2F). This region is believed to occlude the catalytic site upon substrate binding and ranges from several unstructured residues in *B. subtilis* YkuD (Figure 2A), to more complex secondary structures as seen in *M. tuberculosis* Ldt_Mt2_ and *E. coli* LdtD (Figure 2B–D) [58,59,60]. Template modelling scores (TM-scores), ranging from 0–1, where 1.0 indicates identical structure of the catalytic domains of Ldt_Mt2_ and LdtD against YkuD are 0.56 and 0.60, respectively [61]. These are evident of the conserved YkuD fold despite variations in the capping loop and additional secondary structure of the larger Ldt_Mt2_ and LdtD (Figure 2D).

**Figure 3:**
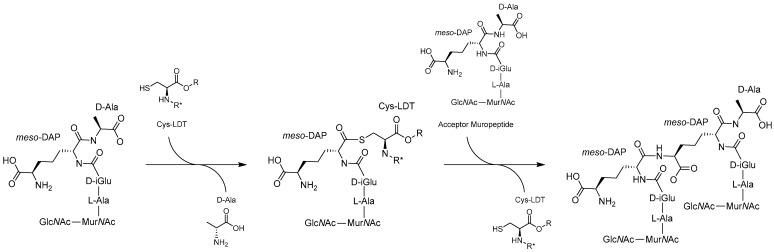
Formation of 3,3-LD-crosslinks as performed by YkuD-type LDTs. The tetrapeptide donor substrate is bound via the L-centre of *meso*-DAP to the catalytic Cys. This displaces the terminal D-Ala allowing the acceptor substrate muropeptide access to the catalytic site. The complex then resolves, forming a 3,3-LD-crosslink between the *meso*-DAP residues of the peptides. R*, R: LDT amino acid chains linking to the catalytic cysteine.

The YkuD mechanism of action starts with the catalytic His abstracting a proton from the catalytic Cys resulting in imidazolium and thiolate ions which act as the general base and nucleophile, respectively [24,57]. The small hydrophobic residue aids in positioning the His near the Cys and accepts an H-bond to stabilise the imidazolium [58,62,64]. Tetrapeptide donor substrate is bound into both cavities where several key residues (e.g., Met303, Tyr308, Tyr318, Thr320, Trp340, His352, and Cys354 in Ldt_Mt2_) position the L-chiral centre of *meso*-DAP near the thiolate [15,65]. This allows the thiolate to perform a nucleophilic attack on the carbonyl carbon forming a reversible tetrahedral acyl-enzyme intermediate (Figure 3) [15,62]. The intermediate is stabilised in an oxyanion hole formed by the backbone NH groups of the Cys and another residue (e.g., Ldt_Mt2_ Cys354/His352 or LdtD Cys528/Tyr507) [15,58,62,65]. The intermediate remains in equilibrium with the unbound state while the terminal D-Ala is released through the outer cavity, allowing the acceptor substrate to enter the active site [20,62,64]. The acceptor is most commonly the D-centre of *meso*-DAP (Figure 3); however, there are many viable substrates including NCDAAs, terminal residues of membrane proteins, and Gly (Table 1) [10,11,13]. The acceptor is positioned proximal to the catalytic His, which acts as the general base catalysing deacetylation and forming the crosslink [15,58,62,65]. In many LDTs like Ldt_Mt2_, the catalytic His is also believed to protonate the leaving group but in some LDTs a Tyr is better positioned to fulfil this role. One example is Tyr507 in LdtD, which is positioned similarly to the mechanism observed in SXN motif PBPs [58,62,66]. This Tyr is not universally conserved in YkuD domains and this role likely varies between species and LDTs [58]. Following transpeptidation the capping loop shifts to uncover the active site allowing the product to be released [58,60,62].

The observed inhibition of this mechanism by β-lactam antibiotics is inconsistent with penicillins and cephalosporins having low affinity while carbapenems and penems have a high affinity [64,69,70]. Studies have found that all β-lactams are bound by LDTs, forming covalent acyl-enzymes; however, the rate of hydrolysis of these complexes is highly variable [64,71]. Penicillin and cephalosporin acyl-enzymes hydrolyse rapidly, becoming inactive and not substantially inhibiting LDTs; alternatively, carbapenem and penem complexes are very stable [56,64,71,72]. This is believed to be due partially to the carbapenems’ and penems’ ability to better mimic the LD-donor substrate [73,74]. It has also been found that the stability of the acyl enzyme complexes depends on the interactions with the capping loop, as carbapenems with smaller side chains, such as imipenem, outperform those with larger sidechains, like meropenem, which can interfere with the capping loop [15,58,59,60,64,65,70,75,76]. The best inhibitor for most LDTs is faropenem, which fragments in the active site, leaving behind a small 86 Da adduct, which allows for greater occlusion by the capping loop [70,77]. The efficacy of carbapenem and penem antibiotics against LDTs is a promising avenue of research, especially in *M. tuberculosis* and *C. difficile* as novel treatments for these pathogens are needed [14,16,77,78,79,80,81,82,83,84].

Many LDTs contain transmembrane (TM) helices, lipid attachment sites, or PG binding domains (Table 2). TM helices are mostly seen in LDTs in Gram-positive bacteria. These anchor LDTs to the CM and may assist during PG synthesis by increasing the proximity to other CM associated PG synthases [16,27,57,73]. Lipoproteins are common in Gram-negative bacteria and Mycobacteria (Table 2) [29,32,33,41]. Those associated with Mycobacteria and Hyphobacteriales are known to contribute significantly to PG structure and cell division. Membrane attachment may allow these LDTs to better associate with other PG synthesis enzymes [32,33,85]. In *Brucella abortus,* membrane attachment is believed to aid in attaching membrane proteins to the PG [41]. This is not consistent in all LDTs involved in protein attachment, for example the specialised LdtA, LdtB, and LdtC in *E. coli* are all predicted to be soluble in the periplasm [10].

Some LDTs contain PG binding domains which aid in localising LDTs to the PG (Table 2). Most common are LysM domains, which are in many PG-associated enzymes; however, LDTs with these domains do not display higher activity than proteins without PG binding domains [24,38,86,87]. For example *E. coli* LdtE is a LysM containing LDT which is active during the stationary phase and stringent response; lpg1386 in *Leigonella pneumophilia* performs a very similar role while lacking a PG binding domain [8,29,68,88]. An exception to this seems to be LDTs carrying the uncharacterised PG-binding domain seen in LdtD and Atu1615 (Table 2) [8,31,32,88]. This domain consists of three α-helices connected by unstructured regions similar to domains observed in phage endolysins and Zn^2+^-amidases [58]. These LDTs are commonly associated with high activity during stress responses, like LPS export stress and unipolar growth disruption [8,32]. Some membrane attached LDTs also contain PG binding domains such as the PG4 domains observed in the redundantly essential Ldt_Cd1_, Ldt_Cd4_, Ldt_Cd5_ in *C. difficile* [16].

**Table 2:** Predicted membrane association of YkuD and VanW family enzymes.

Species	Lipoprotein ^1^	TM Helix	Soluble and PG Binding Domain ^2^	Soluble	Ref.
*M. tuberculosis*	Ldt_Mt2_, Ldt_Mt5_			Ldt_Mt1_, Ldt_Mt3_, Ldt_Mt4_	[29,33]
*B. subtilis*	YciB		YkuD		[24,89]
*E. coli*			LdtC, LdtD, LdtE,	LdtA, LdtB, DpaA	[10,28,43,58]
*C. difficile*		Ldt_Cd1_, Ldt_Cd4_, Ldt_Cd5_		Ldt_Cd2_, Ldt_Cd3_	[16,27]
*E. faecium*		Ldt_fm_			[57]
*A. tumefaciens*	Atu0048, Atu0844, Atu0845, Atu2336, Atu3331, Atu3332, Atu5196		Atu1615, Atu1164, Atu1293, Atu2133	Atu0669, Atu2764, Atu3631	[32,41]
*Dickeya dadantii*			Ldt70, Ldt84	Ldt03, Ldt23	[42]
*Mycobacterium smegmatis*	LdtC_Msm_, LdtB_Msm_, LdtF_Msm_,			LdtA_Msm_, LdtD_Msm_, LdtE_Msm_,	[29,33]
*B. abortus*	Ldt1, Ldt2, Ldt4				[41]
*G. oxydans*	Ldt_Go2_			Ldt_Go1_	[6]
*L. pneumophilia*				Lpg1386, Lpg1336, Lpg0910	[68]
*Coxiella* *burnetii*				Cbu0318, Cbu1138, Cbu0053	[68]

^1^ SEC/SPII or TAT/SPII. ^2^ LysM, PG4, or Phage endolysin/Zn^2+^ amidase.

In parallel to the structural and mechanistic characterisation of members of the YkuD family, investigations were conducted to understand their interactions with β-lactam antibiotics. Initially, these studies focused on LDTs from *M. tuberculosis* as targeting these could generate opportunities to treat infections caused by antimicrobial resistant strains of this pathogen [34,36,62]. It was found that treatment with carbapenems and penems is effective in *M. tuberculosis* as these β-lactams target both PBPs and YkuD-type LDTs, and they are often administered with clavulanic acid, which inhibits the BlaC-type β-lactamases [14,77,78,79,80,90,91]. Various studies have since been conducted to understand how carbapenems interact with LDTs, especially Ldt_Mt2_, the primary LDT of *M. tuberculosis*, with the aim of optimising such treatments [64,65,75,76,81,91,92,93,94,95,96]. It was also discovered that carbapenems could be effective in other species where YkuD-type LDTs contribute to resistance, especially *E. faecium* and *C. difficile* [27,31,58,66,84]. Compound screening has been conducted to discover novel inhibitors of LDTs, while carbapenems remain the last resort antibiotics [82,95,96,97,98].

Furthermore, distinct subgroups within the YkuD family have been identified with specialised roles. Of these the most common is anchoring membrane proteins to PG [10,19,23,32,41]. This most often occurs at the C-terminus of lipoproteins, targeting ε-amino groups of Lys, or the N-terminus of OMPs binding them to *meso*-DAP. Initially identified with LdtA, LdtB, and LdtC in *E. coli*, in recent years many other linkages have been identified in many Gram-negatives (Table 1). Some LDTs only attach membrane proteins (LdtA, LdtB, LdtC) while others have dual functionality with 3,3-TPase activity (Atu0048, Ldt4). Beyond this, YkuD family proteins like DpaA are specialised amidases that detach proteins from the PG [19,43].

Recently it was found that Hyphobacteriales species encode LDTs that specialise in the unique unipolar growth observed in these bacteria which are essential but highly redundant [32,99]. A further subgroup which forms 1,3-LD-crosslinks has been identified, defined by Ldt_Go2_ in *G. oxydans* [6,67] (Table 1). Ldt_Go2_ differs significantly in sequence and structure from typical YkuD-type LDTs, with some researchers classifying them as YkuD-like (Figure 2F) [6,67]. The identification of these differing roles has broadened the study of YkuD LDTs in recent years.

The YkuD family was the only known class of LDTs until the discovery of LD-crosslinking activity of the VanW-type Ldt_Cd4_ and Ldt_Cd5_ in *C. difficile* [16]. These were identified when a *C. difficile* mutant lacking all three YkuD-type LDTs still displayed wild-type (WT) levels of LD-crosslinks in its PG [16]. Alphafold models predict VanW LDTs are membrane attached (Table 1) with putative PG4 PG binding domains and catalytic VanW domains (Figure 2E) [16,63]. The VanW domain contains the same Cys-His-small residue catalytic triad in a similar orientation to YkuD family LDTs despite very different sequences. Beyond this triad the structures bear no similarity and are not superimposable (Figure 2E,F).

## 3. Enterococci Utilise LDTs to Gain β-Lactam Resistance

Ldt_fm_ from *E. faecium* consists of an N-terminal membrane anchor, a predicted pedestal domain, and a C-terminal YkuD catalytic domain with a 19-residue capping loop over the catalytic site [57,66]. Under standard conditions, Ldt_fm_ acts secondary to PBPs and the PG contains ~3% LD-crosslinks [7,17]. β-lactam resistance in *E. faecium* is most commonly associated with an upregulation of the low affinity PBP5 [100,101,102]. However, Ldt_fm_ also has low affinity for penicillins and cephalosporins and LD-crosslinks increase to ~25% under sub-MIC exposure to ampicillin [7,56]. Ldt_fm_-mediated resistance was first discovered in strain M512, which was obtained upon exposure of a strain with a dysfunctional PBP5 D334S variant to ampicillin until an MIC of >2000 µg/mL was achieved [17]. M512 utilises Ldt_fm_ to bypass PBP-mediated PG synthesis, forming a cell wall with 70% LD-crosslinks under standard conditions, increasing to 100% in the presence of β-lactams [7,56]. This PG structure is facilitated by mutations in the *ddcSR* locus and reduction of the phosphoprotein phosphatase activity of StpA. These changes result in production of the cryptic metallo-DD-CPase DdcY and increased Ser/Thr protein phosphorylation, respectively [103,104]. DdcY is essential for generating the tetrapeptide donor peptides of Ldt_fm_ in PG precursors that otherwise contain pentapeptides [103]. It is hypothesised that the reduction in StpA activity allows for the necessary metabolic shifts for high level resistance [104]. Interestingly, a mutation or upregulation of Ldt_fm_ is not required for ampicillin resistance and Ldt_fm_ is believed to become activated based on substrate availability [104]. Strains carrying this resistance mechanism are susceptible to carbapenems, as they can inhibit all PBPs and LDTs in *E. faecium* [30,66,74].

## 4. *Bacillus subtilis* YkuD Defines Its Family but Displays Low Cellular Activity

An LDT of *B. subtilis*, YkuD, is the founding member of the YkuD family of LDTs [24]. X-ray crystallography of YkuD resulted in the first structure of an LDT revealing a C-terminal LysM PG binding domain and the N-terminal catalytic domain, which defines the YkuD-family (Figure 2A) [24,86]. Currently YkuD has no known function and the PG of *B. subtilis* contains a low amount of, if any, LD-crosslinks. NCDAAs are incorporated by PBPs into PG independently of YkuD in *B. subtilis* [13,51,105]. Hence, despite defining the broad family of enzymes, the role of YkuD itself has remained elusive.

*B. subtilis* also contains YciB, a putative lipoprotein with a YkuD-family domain. YciB displays divisome-dependant localisation at mid-cell and the cell poles; however its function has not been explored [89].

## 5. Activity of Two Classes of LDTs Is Essential in *Clostridioides difficile*

*C. difficile* is a Gram-positive bacterium and one of the few known species where LDTs are essential for viability [16]. Its PG consists of ~75% LD-crosslinks, increasing to 85% in the presence of ampicillin [27]. High-level LD-crosslinking is thought to be associated with increased structural integrity of the PG and subsequent resistance to environmental stresses [1,5,27]. *C. difficile* harbours five LDTs, three YkuD family (Ldt_Cd1_, Ldt_Cd2_, Ldt_Cd3_), and two VanW family proteins (Ldt_Cd4_, Ldt_Cd5_) [16,27,72]. Of these, Ldt_Cd1_, Ldt_Cd4_, and Ldt_Cd5_ are redundantly essential for survival [16,27]. Additionally, *C. difficile* spores contain 100% LD-crosslinks; however, only ~1.5% of all peptides are crosslinked and it is not known if they contribute significantly to spore cell wall integrity [84].

The *C. difficile* LDTs have different specialised roles despite their redundancy. Ldt_Cd1_, Ldt_Cd4_, and Ldt_Cd5_ contain PG4 PG-binding domains that are believed to aid in localising the catalytic domain to the PG [16,72]. In vitro testing revealed that Ldt_Cd1_ is the most efficient of the three at incorporating fluorescent TetraRh substrate into purified PG sacculi, but is the least efficient at crosslinking purified tetrapeptide muropeptides [16]. This observation suggests that Ldt_Cd1_ requires a larger substrate than Ldt_Cd4_ or Ldt_Cd5_ [20,72]. Investigation of Ldt_Cd2_ and Ldt_Cd3_ found that, although both display LD-TPase, LD-CPase, and NCDAA substitution activity in vitro, they are not sufficient for viability in vivo [16,20,72]. Interestingly, the purified enzymes also show LD-endopeptidase (LD-EPase) activity, with Ldt_Cd2_ being the most efficient, which has not been observed for other YkuD-type LDTs [20]. This suggests that Ldt_Cd2_ and Ldt_Cd3_ have roles in remodelling PG while Ldt_Cd1_, Ldt_Cd4_, and Ldt_Cd5_ facilitate high level crosslinking during growth and cell division.

These roles are consistent with data from deletion mutants in which the PG composition does not change when one of the essential LDTs is present [16]. Only the depletion of Ldt_Cd5_ in a Δ*ldt_Cd1-4_* strain results in a complete loss of LD-crosslinks and a simultaneous increase in uncrosslinked PG monomers [16,20]. Interestingly, earlier testing of Δ*ldt_Cd1_*, Δ*ldt_Cd2_*, and Δ*ldt_Cd1_ldt_Cd2_* mutants found a significant decrease in LD-crosslinking, by ~35–55%, with a similar increase in monomers [27]. This discrepancy may be due to differences in the background strains used, *C. difficile* R20291 vs. *C. difficile* 630Δ*erm,* respectively [106,107]. Nonetheless, the results indicate that PBPs cannot compensate for the loss of LDT activity in *C. difficile* as there is no change in DD-crosslinking upon the loss of LDTs [16,27].

**Figure 4:**
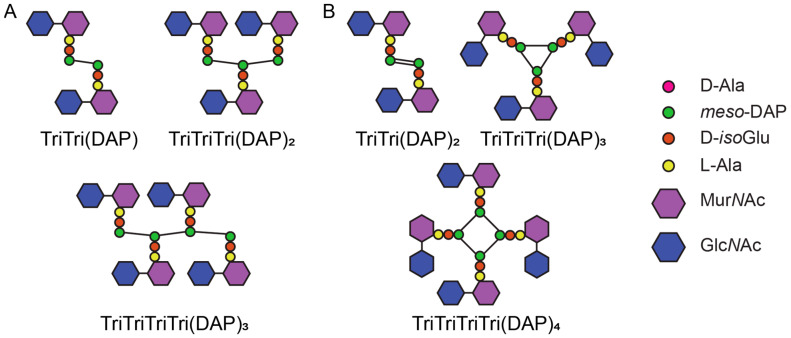
(**A**) Standard structure of LD-crosslinked dimers, trimers, and tetramers. (**B**) Double-crosslinked LD-dimers, cyclic LD-trimers, and LD-tetramers proposed by Galley et al. [20].

The high amount of LD-crosslinks in *C. difficile* PG differentiates it from many species, however this may extend to unique muropeptides as well. Recently, Galley et al. reported double-crosslinked dimers and cyclic trimers and tetramers, with LD-crosslinks formed to both the chiral centres of *meso*-DAP based on mass spectrometry analysis (Figure 4B) [20]. These unusual muropeptides were identified in vitro when testing Ldt_Cd2_ and Ldt_Cd3_. Very low levels (0.05–0.13%) were also detected in PG extracted from cultures [20]. Double crosslinked dimers have not been observed before but given *meso*-DAP has both chiral centres bonded in canonical LD-trimers, this structure is plausible (Figure 4A) [1,11]. The proposed cyclic multimers raise questions about the orientation of the glycan chains to allow the peptides to be in close enough proximity for this structure to form (Figure 4A) [1,108,109].

The reliance on LD-crosslinking in *C. difficile* is of particular interest as it is linked to the high innate β-lactam resistance of this species [110]. This resistance means that *C. difficile* can become the dominant species following β-lactam treatment, causing secondary hospital associated infections [110]. Like other YkuD family LDTs, Ldt_Cd1_, Ldt_Cd2_, and Ldt_Cd3_ can only be inhibited by carbapenems at clinically relevant levels [31,36,64,72]. The VanW family Ldt_Cd4_ and Ldt_Cd5_ are also inhibited by meropenem suggesting a similar mechanism to YkuD LDTs, however their interactions with other β-lactams is unknown [16]. Interestingly, none of these LDTs are directly upregulated during β-lactam exposure indicating constitutive gene expression like in *E. faecium* [20,103,104,106,111]. Upon exposure to sub-MIC levels of ampicillin, *C. difficile* 630Δ*erm* displays an increase in LD-crosslinks accompanied by a decrease in monomers [27]. Conversely, exposure to cefoxitin, meropenem, and imipenem results in a decrease in LD-crosslinks with a subsequent increase in DD-crosslinks and pentapeptide monomers [84]. As the LDTs are not inhibited by cefoxitin the drop in LD-crosslinks may indicate a lack of substrate availability. It may be that cephalosporins disrupt DD-CPases involved in the synthesis of tetrapeptides, which is supported by the increase in pentapeptide monomers [84]. This is of interest as *C. difficile* 630Δ*erm* is resistant to cephalosporins but not carbapenems, suggesting that cephalosporin resistance may not be mediated by LDTs but instead β-lactam resistant DD-CPases and DD-TPases or the intrinsic CDD-2 β-lactamase [84,112]. The mechanism of this resistance is unknown, but likely, *C. difficile* utilises LDTs differently during resistance to penicillins versus cephalosporins [31,84,103,104].

## 6. *E. coli* Requires LDTs for OM Integrity, PG Maintenance, and Stress Responses

*E. coli* contains five YkuD-type LDTs, LdtA (ErfK), LdtB (YbiS), LdtC (YcfS), LdtD (YcbB), and LdtE (YnhG), and a sixth YkuD homolog, DpaA, with PG-Lpp amidase activity. LdtA, LdtB, and LdtC are responsible for anchoring Lpp to the PG, while LdtD and LdtE form LD-crosslinks in the PG [10,28]. None are essential for survival or growth under standard conditions; however, increased membrane permeability and reduced resistance to membrane stress are observed in deletion strains lacking all LDTs [38].

LdtA, LdtB, and LdtC catalyse the attachment of the ε-amino group of the C-terminal Lys of Lpp to the α-carboxyl group of *meso*-DAP in the PG [10,113]. Lpp is the most abundant protein in *E. coli*, anchored to the inner leaflet of the OM, it maintains the spacing between the PG and OM and contributes to the stabilising function of the cell envelope against osmotic challenges [39,114,115]. Deletion mutants lacking LdtA, LdtB, and LdtC display similar phenotypes as Δ*lpp* strains: increased sensitivity to EDTA, SDS, ethidium bromide, and acriflavine, and release of OM vesicles with periplasmic contents [37,38,116,117,118]. Interestingly, the Lpp-PG linkage is dynamic and Lpp can be detached by DpaA, a YkuD-type enzyme which cleaves the L-Lys-*meso*-DAP amide bond [19]. Initially named LdtF, DpaA has an altered active site with only one entrance and lacks an arginine conserved in the catalytic site of LD-TPases. These structural differences allow DpaA to act as a specific PG-Lpp amidase [19,43]. DpaA is not essential under standard growth conditions but becomes essential in cells where LPS export has been disrupted, due to a dysregulation of the PG amidase activator ActS under these conditions [8].

Lpp anchoring has been found to be crucial in mediating the production of the AggR virulence transcription factor in enteroaggregative *E. coli* 042 (EAEC) [119]. Rodriguez-Valverde et al. observed a significant reduction in the level of AggR in an Δ*lpp* strain, displaying reduced biofilm formation and invasion of intestinal colonoids [119]. Reductions in AggR were also seen in Δ*ldtB*, Δ*ldtC*, Δ*ldtBC*, Δ*ldtAB*, and WT cells treated with CuCl_2_, which targets LDTs [120], suggesting this regulation is dependent on Lpp binding to PG [119]. Remarkably, a too tight connection between the PG and OM can be a burden for *E. coli* with an O-antigen-containing LPS upon exposure to bile salts. In this situation, a reduction in OM-PG linkages is beneficial for survival, possibly due to the enlarged periplasmic space that can better accommodate O-antigen which accumulates in the periplasm. Hence, in the human gut, *E. coli* needs to balance its PG-–M interactions to be able to express the O-antigen necessary for gut colonisation, and to survive the bile salts excreted from the gallbladder [121]. Targeting Lpp attachment may provide an opportunity to treat infections with pathogens like EAEC in which Lpp is linked to virulence, such as urinary pathogenic *E. coli* [122], *E. coli* O157:H7 [123], *Salmonella enterica* serovar Typhimurium [124], and *Yersinia pestis* [125].

LdtD and LdtE catalyse the formation of LD-crosslinks in the PG of *E. coli*, but they are expressed under different conditions. LdtE is part of the RpoS regulon and is more highly expressed in stationary phase [8]. It acts as a housekeeping enzyme, reinforcing the PG in the stationary phase and may also have a role in pH stress as its LD-TPase activity at low pH is greater than that of LdtD [88].

LdtD is part of the Cpx regulon, which is activated in response to cell envelope stress [126,127]. LdtD is also upregulated during cold shock, low pH, DNA damage, LPS export disruption, and β-lactam stress [8,31,59,128,129,130]. Its activity is significantly above that of LdtE at neutral pH both in vivo and in vitro [88]. LdtD is further regulated by the RNA helicase DeaD, which unravels the *ldtD* mRNA secondary structure, increasing translation. This regulation was identified in conjunction with cold shock, however, it is likely that it extends beyond this condition [128]. DD-CPases also regulate LdtD activity through substrate availability under standard and stress response conditions [8,31,131,132]. Overall LdtD is a high activity stress response LDT regulated at transcriptional, translational, and substrate levels.

LdtD is not essential under standard conditions, however, it becomes essential during severe envelope stress when LPS export is disrupted and in the β-lactam resistant strain M1 [8,31]. Under these conditions several other PG enzymes also become essential and likely form a complex to bypass canonical DD-TPase based PG synthesis [8,31,58]. These enzymes are the glycosyltransferase (GTase) domain of PBP1B with its activator LpoB, and a DD-CPase either PBP6B during LPS export stress or PBP5 for β-lactam resistance [8,31,58]. It was proposed that a block in LPS export results in defects in the PG due to disassembly of the envelope-spanning Lpt machine and these defects need to be repaired to prevent cell lysis [8,133]. For PG repair, LpoB-activated GTase of PBP1B forms glycan chains before DD-CPases remove the terminal D-Ala of the pentapeptides, forming nascent PG with tetrapeptides, which are crosslinked to peptides in the sacculus by LdtD [8,31,58]. In support of this model, LdtD is known to interact with PBP1B in vitro and in vivo during LPS export disruption [8]. Furthermore, modelling data suggests that the apolar scaffold domain of LdtD interacts with apolar regions of the GTase domain of PBP1B and the PBP5 hinge domain positioning its catalytic site to crosslink the nascent glycan strands [58]. In the β-lactam resistant M1-M7 strains, the elevated level of (p)ppGpp compensates for the canonical elongation and cell division machineries [31]. Analysis of the PG of M1 revealed a 100% LD-crosslinking when grown in the presence of ampicillin, suggesting canonical DD-TPases had been completely inactivated and are bypassed [31]. This condition requires LD-EPases to facilitate the hydrolysis of PG crosslinks necessary for growth and cell division [131,134]. LD-EPase activity can be provided by the LD-specific EPase MepK, or overexpression of the DD/LD EPases MepA and MepS if MepK is deleted [131]. Like most YkuD family LDTs, LdtD has low affinity for penicillins and cephalosporins but is susceptible to carbapenems [31,58,59]. Much remains to be known about LdtD and its roles within *E. coli,* especially given its complex regulation.

## 7. An LDT Is Essential for Typhoid Toxin Release in *Salmonella enterica* Serovar Typhi

In *S. enterica* serovar Typhi, LdtD (YcbB) is required for the release of typhoid toxin, the main virulence factor responsible for typhoid fever [45]. *S. enterica* serovar Typhi is an intracellular pathogen which invades host cells and resides in modified vacuoles where it replicates and releases typhoid toxin [135]. However, typhoid toxin is a large protein complex which cannot pass through the periplasmic PG layer via diffusion. LdtD is expressed when Salmonella grows within a host cell, specifically modifying the PG with LD-crosslinks. Typhoid toxin then localises at the pole accompanied by the LD-specific EPase TtsA. TtsA is encoded in the typhoid pathogenicity island and hydrolyses LD-crosslinks, locally increasing the pore size in the PG and facilitating toxin release [45]. It is unclear if this process requires DD-EPase activity to cleave the existing crosslinks in the PG or whether LdtD activity is sufficient to facilitate TtsA-mediated toxin translocation. The use of PG hydrolases to mediate the export of a large protein (complex) have been termed as type 10 secretion systems (T10SS) [45,46]. Another T10SS relies on the L-Ala-D-isoGlu endopeptidase ChiX, which is required for Chitinase secretion in *Serratia marcescens* [136,137].

## 8. LDTs Facilitate Environmental Adaptation in *Vibrio* Species

*Vibrio cholera* contains two LDTs, the LD-TPase LdtA and the Lpp-PG attaching LdtB, both also incorporate NCDAAs into the PG [13]. This activity promotes changes in PG synthesis to maintain the necessary amount and strength required as cells enter stationary phase [12]. In *V. cholera* NCDAA incorporation is controlled by RpoS with endogenous NCDAAs being produced by the racemase BsrV during the stationary phase [12,13]. Alternatively, exogenous NCDAAs can be incorporated, prompting similar changes [13]. This variation allows *V. cholera* to alter its PG depending on colony state or environmental influences when growing in a community [12]. This process is also observed in other bacteria with some relying on LDTs and others utilising PBPs for NCDAA incorporation [13,51,138]. Incorporation of NCDAAs allows cells to react to metabolic or community changes increasing survival [51,138].

*Vibrio fischeri* utilises LdtA to increase its fitness during the colonisation of the bioluminescent crypts of the bobtail squid [47]. *V. fischeri* is naturally bioluminescent and associates symbiotically with bobtail squid to provide camouflage in the night-time ocean [47]. The association is cyclic, with 95% of the *V. fischeri* bacteria expelled from the three bioluminescent crypts every morning, only to repopulate by dusk. The crypts have different sizes and the bacteria grow at different rates in each [47]. LdtA expression correlates with the slower growth stages in the larger crypts and is constitutive in the smaller ones. It was found that a Δ*ldtA* strain is consistently outcompeted by the WT during crypt recolonisation despite both growing similarly in broth cultures [47]. This suggests that LdtA is important in the symbiosis stress response, possibly strengthening the PG or increasing resistance to immune lysozymes [47].

## 9. LDTs Mediate a Wide Range of Membrane-PG Attachments Within γ-Proteobacteria

**Figure 5:**
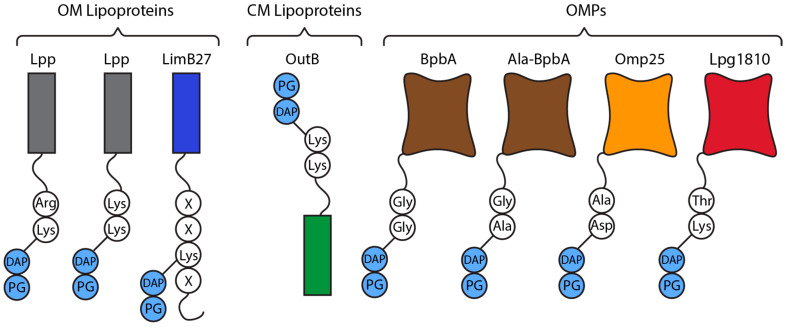
Representative membrane lipoproteins and OMPs tethered to PG (light blue) and their attachment sites (white). *E. coli* Lpp (grey) C-terminal Lys-Arg, *D. dadantii* Lpp C-terminal Lys-Lys, *C. burnetii* LimB27 (dark blue) Lys_21_, *D. dadantii* OutB C-terminal Lys-Lys, *C. burnetii* BpbA (brown) N-terminal Ala-Gly, *C. burnetii* BpbA N-terminal Gly-Gly, *B. abortus* OMP25 (orange) N-terminal Asp-Ala, *L. pneumophilia* Lpg1810 (red) C-terminal Lys-Thr.

The attachment of Lpp is the best understood LDT-mediated OM anchoring mechanism, however, Lpp is not conserved throughout the γ-proteobacteria. In other bacteria with or without Lpp, LDTs can attach other OM proteins to PG (Figure 5). The links between the PG and OM maintain the integrity of the OM with OM dissociation, vesiculation, and increased OM permeability seen whenever the PG-OM attachment is disrupted [23,37,42,121].

Species like *Coxiella burnetii* and *L. pneumophilia*, the causative agents of Q-fever and legionnaires disease, respectively, lack Lpp and instead rely on unique lipoproteins and OMPs to anchor the OM to the PG [23]. *C. burnetii* contains two OMPs (BpbA and BpbB) with transmembrane β-barrel structures and glycyl-glycyl motifs at the N-terminus in the periplasmic part [23]. The Gly-Gly motif can be attached to the PG directly by Ldt2 which forms Gly-Gly-*meso*-DAP linkages (Figure 5). BpbA also forms Gly-Ala-*meso*-DAP attachments through a currently unknown mechanism (Figure 5) [23]. The glycyl-glycyl N-terminal motif is conserved in OMPs from many γ-proteobacteria species, often observed in species with many LDT homologs [23]. *C. burnetii* and *L. pneumophilia* contain ten and eleven YkuD family LDTs, respectively. Species with only a few LDTs, like *V. cholera*, *E. coli*, and *Pseudomonas aeruginosa*, are not known to form these linkages [23].

*C. burnetii* also displays the *Coxiella* specific LimB27, an OM lipoprotein which is tethered by its Lys_21_ residue to *meso*-DAP by an unknown LDT (Figure 5) [23]. The combination of BpbA, BpbB, and LimB27 holds the *C. burnetii* OM ~35 Å away from the PG, a distance that is much closer than that of *E. coli* (~85 Å) [23]. The importance of this difference is currently unknown.

Recently it was discovered *L. pneumophilia* also attaches Lpg1810, an OM long chain fatty acid transporter, to the PG by its C-terminal Lys-Thr motif (Figure 5) [68]. This tethering was only observed in nutrient limited tap water conditions suggesting Lpg1081 fulfills a unique role in these conditions [68]. Lpg1810 homologs have been identified throughout Proteobacteria [68].

*D. dadantii* is a γ-proteobacterium and plant pathogen, which contains a unique LDT mediated CM protein-PG linkage [42]. Its OM is linked to PG via Lpp with *D. dadantii* utilising a C-terminal Lys-Lys instead of the Lys-Arg in *E. coli* [10,39,42]. *D. dadantii* links its CM to the PG via OutB (Figure 5) [42]. OutB is a scaffold protein for the Out system and consists of an N-terminal transmembrane domain, an Out system homology region, and a C-terminal end (VRTTKK) which mimics that of Lpp (VRTYKK) (conserved residues underlined) [42]. This C-terminal region allows both proteins to be anchored by two LDTs with semi redundant activity, Ldt03 and Ldt84 [42]. Despite being part of the Out system which assembles the outer membrane protein OutD, OutB expression is independent. This allows its upregulation during plant infection which indicates a role in reinforcing the cell envelope during invasion [42].

## 10. LDTs Are Central to the Formation of Antibiotic-Resistant Variants in *C. burnetii* and *L. pneumophilia*

The intracellular pathogens *C. burnetii* and *L. pneumophilia* form two distinct cell morphologies, with intracellular ‘replicating variants’ and infectious ‘survival variants’ [68]. Survival variants are resistant to many antibiotics and environmental factors allowing them to act as the main infection vectors for these species. The transition between forms is controlled via the RpoS regulon as the cultures enter stationary phase, which induces PG remodelling by various LDTs among other adaptations. In both species, DD-crosslinking drops from ~7% to ~3% while LD-crosslinking increases from negligible levels to ~10% [68]. This response is enhanced when *C. burnetii* is cultured under intracellular conditions, with more crosslinking and OM tethering in this environment [68]. Each species has five LDTs that are upregulated during the RpoS response, with one main LDT upregulated to a higher degree. Deletion of the most upregulated LDT gene in *L. pneumophilia*, *lpg*1386, increases antibiotic susceptibility and reduces survival in nutrient limited tap water [68]. This is accompanied by a significant decrease in LD-crosslinking, however, it is not abolished, indicating a level of redundancy between LDTs [68]. This finding is parallelled in other species with multiple LDTs where specialisation is observed alongside apparent redundancy [16,32].

## 11. LDTs Specialised in Polar Growth and OMP-PG Attachment Are Essential for Viability in Hyphobacteriales

**Figure 6:**
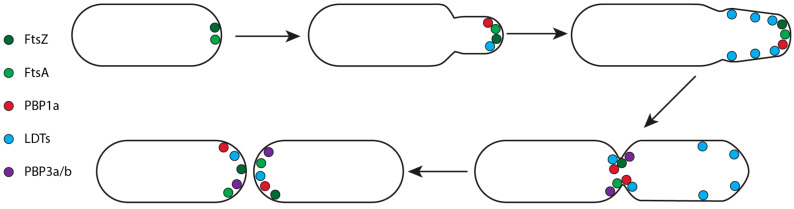
Representation of the localisation pattern of PG synthases during unipolar division of *A. tumefaciens*.

Members of the order Hyphobacteriales (Rhizobiales) are α-proteobacteria living in the soil or on plants. They are rich in YkuD family LDTs, commonly containing 5–8 homologs with up to 21 being observed [99]. Hyphobacteriales are characterised by their unipolar growth where the cell envelope is synthesised near one of the poles, forming a new small ‘chamber’ which increases in length and diameter before septal formation at the midcell initiates cell division and daughter cell separation (Figure 6) [139,140,141]. Hyphobacteriales species also utilise OMPs to anchor their OM to the PG via conserved N-terminal alanyl-aspartyl motifs (Figure 5) [23,41].

The unipolar growth of Hyphobacteriales requires the class A PBP1a and at least one LDT to initiate and expand the new chamber [32,140]. PBP1a is required to insert new PG at the cell pole, localising after FtsA/FtsZ and filling the role of the RodA-PBP2 complex which is lacking in Hyphobacteriales (Figure 6) [99,139,140]. Simultaneously, LDTs localise at the cell pole early in the cell cycle, assisting in PG insertion [32,99,139]. As the cycle progresses their activity diffuses to the lateral wall of the new chamber to facilitate elongation and an increase in cell diameter (Figure 6) [99,140]. Eventually, the polar growth machinery dissociates, localising at the midcell alongside PBP3a and PBP3b to facilitate septum formation and cell separation (Figure 6) [99,139,140].

Phylogenetic analysis of the 14 LDTs in *A. tumefaciens* found those strongly associated with polar growth are part of a subgroup of YkuD family LDTs unique to Hyphobacteriales [32,99]. Three members of this group, Atu0048, Atu0845, and Atu331, are sufficient for viability as the sole LDT in the absence of all other LDTs [32]. A Δ13LDT mutant with Atu0048 as the sole LDT most closely resembles WT in growth and cell morphology, Atu0048 also robustly localises to the poles and likely acts as the main LDT during unipolar growth [32,99]. Interestingly, the Hyphobacteriales subgroup LDTs are not essential for survival as all seven can be successfully deleted resulting in Δ7LDT strains which display less polar LDT activity, slowed growth, and osmotic sensitivity [32]. In this Δ7LDT strain Atu1164, an LdtD homolog, becomes essential. Given the pattern of LDT activity in these strains, it is likely Atu1164 specialises in elongation and expansion, with PBP1a facilitating polar growth [32]. This demonstrates that the LDTs of *A. tumefaciens* have specialised roles under standard conditions despite displaying functional redundancy [32].

Disruption of OMP-PG linkages in Hyphobacteriales results in OM blebbing at high temperatures consistent with OM instability [37,41,118]. In *B. abortus* two OMPs (OMP25 and Omp2b) are redundantly attached by Ldt1, Ldt2, and Ldt4 [41]. These are the only three putative lipoproteins of the eight LDTs in *B. abortus*, and the lipid modification might assist in localisation to the OM [41]. Deletion of these LDTs alone or in tandem resulted in increased OM blebbing at higher temperatures, however the periplasmic width remained unchanged [41]. As *B. abortus* contains seven PG attached OMPs and eight LDTs it is likely that there is greater redundancy in attachments than those covered in the initial study [41]. Similarly, *A. tumefaciens* has six PG tethered OMPs attached by its 14 LDTs [32,41]. Studies of three of these OMPs, AopA1 (RopA1/*atu1020*), AopA2 (RopA2/*atu1021*), and AopB (RopB/*atu1131*), gave insights into the redundancy of this system [32,41]. Deletions of different LDTs resulted in attachments shifting ~10-fold up or down depending on the LDTs targeted [32]. The LDTs favour attaching OMPs with similar structures as the closely related AopA1 and AopA2 have similar attachment levels in any given mutant while AopB attachment varies independently [32]. This is clearly seen in Δ13LDT mutants as Δ13LDT Atu0048 appears incapable of attaching AopB while AopA1/AopA2 increases slightly [32]. Meanwhile Δ13LDT Atu0845 only increases AopB while AopA1/AopA2 remain steady and Δ13LDT Atu3331 increased all three OMPs attachment by 10-fold [32]. In summary, the diversity of OMPs attached to PG by multiple LDT homologs generates a complex OM-PG tethering system in Hyphobacteriales.

## 12. A Novel Class of LDTs Forms 1,3 LD-Crosslinks in α- and β-Proteobacteria

**Figure 7:**
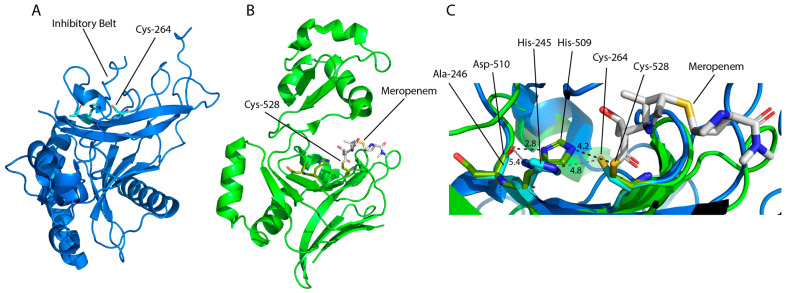
(**A**) Cartoon structure of YkuD like catalytic domain of *G. oxydans* Ldt_Go2_ (PDB: 8QZG res 52–331) [6]. (**B**) Cartoon structure of YkuD catalytic domain of LdtD (PDB: 6NTW res 375–576) [58]. (**C**) Comparison of the key catalytic triads of Ldt_Go2_ and LdtD, Ldt_Go2_ autoinhibited by belt structure as seen by Ala246-His245 distance, LdtD Cys-528 covalently inhibited by meropenem. TM-value of the Ldt_Go2_ catalytic domain alignment with LdtD catalytic domain was 0.37 [61].

Recent studies in *G. oxydans* identified two YkuD family LDTs, Ldt_Go1_ (GOX2269) and Ldt_Go2_ (GOX1074), one of which, Ldt_Go2_, is capable of forming 1,3 LD-crosslinks. Homologs have been identified across α- and β-proteobacteria, mostly Acetobacteriaceae and Burkholderiaceae. These LDTs are phylogenetically unique from each other and other known YkuD family LDTs, with Ldt_Go2_ being categorised as YkuD-like (Figure 2F) [6,67]. A comparison of the crystal structures of Ldt_Go2_ and LdtD (TM-score 0.36) shows structural differences in the catalytic domain, with β5-β6 being longer and connected by an extended loop, similarly β3–β4 being connected by an extended loop (Figure 7A,B) [6]. These structural changes give Ldt_Go2_ a wider catalytic site which might assist the binding of the L-Ala, which resides in close proximity to the glycan chain [6]. Interestingly, Ldt_Go2_ may be autoinhibited by a ‘belt’ structure which occludes the catalytic site and prevents a Cys–His interaction (Figure 7C) [6]. For Ldt_Go1_ there is no activity or structural data available so far [67].

1,3-LD-crosslinking was first observed in various species of Acetobacteriaceae and was identified as providing lysozyme resistance during the stationary phase [48]. These crosslinks increase from ~5.6% in exponential phase to 16.6% in the stationary phase [48,67]. Deletion of Ldt_Go2_ eliminated 1,3-LD-crosslinks and they were restored upon complementation showing that Ldt_Go2_ is required for their formation [6,67]. Furthermore, the heterologous expression of Ldt_Go2_ homologs from multiple species in *E. coli* resulted in the production of 1,3-LD-crosslinks [6]. Deletion of *ldt_Go1_* yielded different results. Espaillat et al. observed a PG profile comparable to that of WT while Alamán-Zárate et al. observed a ~50% decrease in 1,3-LD-crosslinking [6,67]. This difference may be due to the use of different parental strains with varying LDT redundancy.

In vitro testing of Ldt_Bcn_, an Ldt_Go2_ homolog from *Burkholderia cenocepacia*, found that it utilises donor muropeptides with tri- and tetrapeptides and preferentially utilises monomers [6]. This preference explains why the deletion of the gene encoding the DD-EPase PBP7 results not only in an increase in DD-crosslinks, but also a decrease in 1,3-LD-crosslinks [6]. 1,3-LD-crosslinks have been shown to protect *G. oxydans* against lysozymes (muramidases) and it is hypothesised that they strengthen the PG allowing *G. oxydans* to withstand the low pH, high ethanol, and high temperature conditions Acetobacteriaceae are known for [6,48,67,142].

## 13. Mycobacterial LDTs Produce High-Level Crosslinking to Strengthen PG and Resist Environmental and Antibiotic Stress

Mycobacteria possess highly crosslinked PG consisting of both DD- and LD-crosslinks. For example, *M. tuberculosis* has 70–80% of available peptides crosslinked, with 30–60% being LD in exponential phase with up to 80% reported in stationary phase [5,143,144,145,146]. These reports are varied as traditional techniques for preparing and analysing PG are ineffective in Mycobacteria due to the inability to quantitatively remove the arabinogalactan and mycolic acids [11]. Despite this it is clear that LDTs play a significant role in Mycobacteriales, *M. tuberculosis* and *M. abscessus* have five YkuD family homologs, while *M. smegmatis* has six [29,34,146].

*Mycobacterial* LDTs are divided into six classes based on structural homology of 323 LDTs across 83 genomes (Table 3) [29,33]. All classes contain the YkuD-type catalytic domain and one or two immunoglobulin like BIg-5 structural domains. Further differentiation is based on variations in C-terminal subdomains, N-terminal lipoboxes, proline rich regions, and insertions in the catalytic domain [33]. Classes one, two, five, and six demonstrate LD-TPase activity in vitro and in vivo, whereas class three and four activity has only been observed in vitro [34,143,147,148].

In *M. tuberculosis*, the class two Ldt_Mt2_ is the most active LD-TPase, expressed 10-fold more than other LDTs during exponential growth phase with high activity near neutral pH [34,147]. Ldt_Mt2_ contains a canonical YkuD domain with two BIg-5 domains [33]. Deletion of *ldt_Mt2_* results in smaller colonies, reduced virulence and susceptibility to amoxicillin and clavulanate or vancomycin [34,92]. Electron microscopy identified unusual cell morphologies with decreased cell length, surface defects, and irregular cell widths [92]. These reports have made Ldt_Mt2_ a key target for potential antibiotics as its inhibition alongside classical PBPs is lethal [80].

The class one Ldt_Mt1_ and class five Ldt_Mt5_ have been observed to maintain PG structure and integrity during the stationary phase and stress responses in *M. tuberculosis* [143,147]. Ldt_Mt1_ is upregulated 17-fold during starvation responses and increases LD-crosslinks in the stationary phase [143]. Ldt_Mt5_ has been linked to stress responses, as it has highest activity at pH 9, and is linked to crystal violet and osmotic shock resistance [147]. Mutants lacking Ldt_Mt1_ or Ldt_Mt5_ have limited growth or morphology defects but their absence alongside Ldt_Mt2_ exacerbates the Δ*ldt_Mt2_* phenotype, indicating partial redundancy [92]. Δ*ldt_Mt2_*Δ*ldt_Mt1_* and Δ*ldt_Mt2_*Δ*ldt_Mt5_* mutants grow slower and show increased susceptibility to amoxicillin/ampicillin and clavulanate or vancomycin, as well as increased surface and cell wall defects [92]. Class five LDTs are unique among YkuD family TPases as they are not inhibited by carbapenems. The mechanism of this low affinity is currently unknown, but it reinforces the need for LDT specific antibiotics [29,85,148].

The class three and four LDTs have no identified roles in *M. tuberculosis* or any other Mycobacteriales species. Both are acylated by β-lactams and purified Ldt_Mt4_ forms LD-crosslinks from tetrapeptide substrates [148,149]. The N-terminal proline rich region (PRR) present in Ldt_Mt4_ is a motif often associated with protein–protein interactions and/or disordered regions, which may indicate that Ldt_Mt4_ binds an activator or its acceptor substrate via this region [33]. It may be that these classes have roles in PG modification or adaptation in hostile environments during infection and colonisation.

The class six LDTs, which are absent from *M. tuberculosis*, are structurally similar to class two LDTs with a minor C-terminal domain and a 10-residue insertion in their catalytic domain. They are believed to perform similar roles to class two and class five, although they might be able to act on larger substrates [29,33].

The LDTs of *M. smegmatis* follow a similar pattern to those of *M. tuberculosis* with one major LD-TPase, LdtC_Msm_, and two secondary LD-TPases, LdtB_Msm_ and LtdF_Msm_. A Δ5LDT_Msm_ LdtC+ strains resemble WT, while Δ6LDT_Msm_ strains lose cell shape with notable membrane blebbing and division inhibition, pointing to important roles of LdtC_Msm_ in morphogenesis and membrane integrity [85,150]. Consistent with this conclusion, a Δ*ldtC* strain displays increased susceptibility to rifampicin, ampicillin, imipenem, lysozyme, and D-methionine, reinforcing its contribution to cell wall integrity [29,150]. LdtB_Msm_ and LtdF_Msm_ have partially redundant functions as the defects of Δ*ldtC* are exacerbated in Δ*ldtBCF* and the Δ6LDT_Msm_ mutants. In isolation, the lack of LdtB_Msm_ or LtdF_Msm_ are linked to low level rifampicin sensitivity, indicating supporting roles in maintaining membrane integrity [150]. Antibiotic testing with various β-lactams correlated in vitro LDT and PBP inhibition with MICs in *M. smegmatis*. Carbapenems and penems display high efficacy while cephalosporins and penicillins, which have low affinity to LDTs, were ineffective [151]. Interestingly, novel Ldt_Mt2_ inhibitors had little efficacy against *M. smegmatis*. This may be due to lack of inhibition of the essential PBP3 [151]. This indicates that inhibition of both PBPs and LDTs of *M. smegmatis* is necessary for efficient antimycobacterial drugs [151].

Since the discovery of the importance of LDTs in Mycobacteria, they have become a target for new treatments of these notoriously resilient bacteria [14]. β-lactams have rarely been used for targeting *M. tuberculosis* because of its β-lactamase (BlaC); however, with the advent of β-lactamase inhibitors such as clavulanic acid this treatment route may be viable [14,80]. Combination therapies have been trialled with carbapenems + clavulanic acid [80,90] or β-lactamase resistant faropenem and rifampin [78]. In vitro testing revealed that these combinations are more effective against multidrug resistant strains than susceptible strains [78]. This may be because resistance mechanisms often reduce fitness under standard conditions [78]. While these advances are promising further research is required if these treatment options are to be utilised.

## 14. LDTs Incorporate Probes for Microscopy

LDTs have been utilised for many years to facilitate PG specific microscopy probes for both electron and fluorescent microscopy. First used by de Pedro et al., the incorporation of D-Cys into the PG of *E. coli* allowed pulse chase experiments to investigate where the nascent PG is inserted during the cell cycle. Purified sacculi with incorporated D-Cys were biotinylated at the sulfhydryl groups, followed by Protein A-gold immunolabelling and visualisation by transmission electron microscopy (TEM) [49]. The spatial incorporation pattern of D-Cys allowed for quantification of PG segregation and expansion during growth [49]. This technique has been adapted for both fluorescent and electron microscopy in investigations of PG growth, adaptation to environmental stresses, and the effects of antibiotics on sacculus growth [152,153,154,155].

D-Cys biotinylation requires purification of PG sacculi, preventing real-time imaging of growing cells. This need led to the development of the fluorescent D-amino acid probes (FDAAs), such as HADA and NADA, which can be incorporated into the PG of actively growing and dividing cells [50]. These probes are incorporated by either LDTs or PBPs, allowing their use in investigating PG throughout the cell cycle of many species [13,50]. FDAAs rely on the substitution reaction (Figure 1) and are important tools to specifically label the sites of TPase activity in cells [51].

Pidgeon et al. developed further probes for LDT and PBP TPase activity [156]. They synthesised peptides which mimic tetrapeptide and pentapeptide substrates with a fluorophore in the position of Mur*N*Ac [52,156]. Using these probes they were able to distinguish between LD-TPase and DD-TPase activity and they demonstrate how each is affected by different antibiotics [156]. More recent work by Walenkiewicz generated multiple dipeptide versions of the LD-specific probes consisting of D-Ala-L-Lys-fluorophore, with different fluorophores replacing D-*iso*Gln in the peptide [52]. These probes were incorporated into the cell wall of several species, even those which contain *meso*-DAP instead of L-Lys. Their incorporation was consistent with the known LDT activity patterns such as the cell poles in *A. tumefaciens* [52]. Further development of these probes to incorporate *meso*-DAP would make them more representative of native conditions in species which have *meso*-DAP in their PG [52]. The development of small and efficient LD-TPase probes has generated opportunities for understanding of the cellular roles of these enzymes.

## 15. Conclusions

The two families of LDTs, YkuD and VanW, are conserved in many bacteria and they have different activities and roles. The majority function as 3,3-LD-TPases forming crosslinks in the PG, which is believed to increase its strength [2,27,52]. LDTs have roles in essential stages of the cell cycle, stress response, or remodelling PG during the stationary phase [5,8,27,56]. They contribute to β-lactam resistance in pathogens such as *M. tuberculosis*, *E. faecium,* and *C. difficile*, all of which can utilise LDTs to avoid killing by penicillins or cephalosporins [7,14,27,31,84]. The β-lactam resistance mechanisms are diverse and often combine increased LDT activity with changes to key metabolic pathways to facilitate substrate availability [84,103,104]. The diverse YkuD family proteins also attach lipoproteins and OMPs to PG, adding to the diverse roles of LDTs [23,32,41,42,43]. Furthermore, YkuD LDTs catalyse the formation of 1,3-LD-crosslinks [6,67], 3-3-crosslinks are used by T10SSs [45], and the regulation of virulence factors [119] has been linked to this family. The discovery of the LDT activity of VanW family of proteins opens new research directions understanding their roles in different species and uncovering the activity of the non-LDT homologs [16,26].

As our understanding of LDTs increases it is clear that their roles range widely between and even within species. Their ability to target various substrates, catalyse different reactions, and act in conjunction with other PG enzymes provides many future directions for study. This is only furthered by their interactions with certain β-lactam antibiotics which may provide ways to treat severe infections by *M. tuberculosis*, *C. difficile*, and more. It is clear that LDTs have a major contribution to the unique plasticity of the PG and how bacteria adapt to various conditions.

## Figures and Tables

**Table 1:** Summary of LDT activity in different species.

Activty	Product	LDT	Species	Ref.
3,3-LD-TPase	*meso*-DAP-*meso*-DAP	LdtD	*E. coli*	[28]
	L-Lys-D-Axn-L-Lys	Ldt_fm_	*E. faecium*	[56]
1,3-LD-TPase	L-Ala-*meso*-DAP	Ldt_Go2_	*G. oxydans*	[6,67]
NCDAA Substitution	NCDAA-*meso*-DAP/L-Lys	LdtA/B	*V. cholera*	[13]
Lpp Attachment	Lpp-Lys-Arg-*meso*-DAP	LdtA/B/C	*E. coli*	[10]
	Lpp-Lys-Lys-*meso*-DAP	Ldt03/84	*D. dadantii*	[42]
OutB Attachment	OutB-Lys-Lys-*meso*-DAP	Ldt03/84	*D. dadantii*	[42]
LimB27 Attachment	Lys_21_-*meso*-DAP	unknown	*C. burnetii*	[23]
OMP Attachment	Gly-Gly-*meso*-DAP	Ldt2	*C. burnetii*	[23]
	Gly-Ala-*meso*-DAP	unknown	*C. burnetii*	[23]
	Ala-Asp-*meso*-DAP	Ldt4	*B. abortus*	[41]
	Thr-Lys-*meso*-DAP	unknown	*L. pneumophilia*	[68]
Lpp Detachment	Lpp-Lys-Arg + *meso*-DAP	DpaA	*E. coli*	[19]
LD-CPase	L-Ala + *meso*-DAP	Ldt_Cd2_	*C. difficile*	[20]
LD-EPase	*meso*-DAP + *meso*-DAP	Ldt_Cd2_	*C. difficile*	[20]

**Table 3:** Summary of Mycobacterial LDT classes as described by Zandi et al. [33].

Class	Catalytic Domain	Structural	Additional Regions	Examples
1	YkuD + Region 1	Big ^1^		Ldt_Mt1_, LdtA_Msm_
2	YkuD	2 BIg	Lipoprotein ^2^ C-terminal domain	Ldt_Mt2_, LdtB_Msm_
3	YkuD + Region 1 ^3^ + Region 2 ^4^	BIg		Ldt_Mt3_, LdtD_Msm_
4	YkuD	BIg	N-Terminal PRR ^5^ C-terminal domain	Ldt_Mt4_, LdtE_Msm_
5	YkuD	2 BIg	Lipoprotein 2 C-terminal domains	Ldt_Mt5_, LdtC_Msm_
6	YkuD + 10 Res insertion ^6^	2 BIg	lipoprotein C-terminal domain	LdtF_Msm_

^1^ Big—Bacterial Ig-like domain. ^2^ Characterised by an N-terminal lipobox. ^3^ Conserved sequences in the Catalytic domains of class one and three. ^4^ Variable length sequences rich in proline and glutamate. ^5^ PRR- Proline Rich Region. ^6^ 10 residue insertion between β-sheets 20 and 21 near the active site.

## Abbreviations

The following abbreviations are used in this manuscript:

BIg	Bacterial Immunoglobulin-like domain
CM	Cytoplasmic Membrane
CPase	Carboxypeptidase
EPase	Endopeptidase
FDAA	Fluorescent-Labelled D-Amino Acid
Glc*N*Ac	*N*-Acetylglucosamine
GTase	Glycosyltransferase
LDT	LD-Transpeptidase
*meso*-DAP	*meso*-Diaminopimelic Acid
MIC	Minimum Inhibitory Concentration
Mur*N*Ac	*N*-Acetylmuramic acid
NCDAA	Non-Canonical D-Amino Acids
OM	Outer Membrane
OMP	Outer Membrane Protein
PBP	Penicillin-Binding Protein
PG	Peptidoglycan
PRR	Proline-Rich Region
TEM	Transmission Electron Microscopy
TM	Transmembrane
TM-score	Template Modelling score
TPase	Transpeptidase
T10SS	Type 10 Secretion System
